# Intracellular Cleavage of Amyloid β by a Viral Protease NIa Prevents Amyloid β-Mediated Cytotoxicity

**DOI:** 10.1371/journal.pone.0098650

**Published:** 2014-06-10

**Authors:** Baehyun Shin, Hyejin Oh, Sang Min Park, Hye-Eun Han, Michael Ye, Woo Keun Song, Woo Jin Park

**Affiliations:** 1 School of Life Sciences, Gwangju Institute of Science and Technology, Gwangju, Korea; 2 Bio Imaging and Cell Dynamics Research Center, School of Life Sciences, Gwangju Institute of Science and Technology, Gwangju, Korea; 3 Center for Human Genetic Research, Massachusetts General Hospital, Boston, Massachusetts, United States of America; University of the Witwatersrand, South Africa

## Abstract

Nuclear inclusion a (NIa) of turnip mosaic virus is a cytosolic protease that cleaves amyloid β (Aβ) when heterologously overexpressed. Lentivirus-mediated expression of NIa in the brains of APP(sw)/PS1 mice significantly reduces cerebral Aβ levels and plaque depositions, and improves behavioral deficits. Here, the effects of NIa and neprilysin (NEP), a well-known Aβ-cleaving protease, on oligomeric Aβ-induced cell death were evaluated. NIa cleaved monomeric and oligomeric Aβ at a similar rate, whereas NEP only cleaved monomeric Aβ. Oligomeric Aβ-induced cytotoxicity and mitochondrial dysfunction were significantly ameliorated by NIa, but not by NEP. Endocytosed fluorescently-labeled Aβ localized to mitochondria, and this was significantly reduced by NIa, but not by NEP. These data suggest that NIa may exerts its protective roles by degrading Aβ and thus preventing mitochondrial deposition of Aβ.

## Introduction

Alzheimer’s disease (AD) is the most prevalent neurodegenerative disorder that is characterized by progressive memory impairment and cognitive dysfunction. The hallmarks of AD are the formation of intracellular neurofibrillary tangles composed of hyper-phosphorylated tau and extracellular amyloid plaques mainly composed of amyloid β (Aβ). Aβ is generated through sequential cleavage of amyloid precursor protein (APP) by β- and γ-secretases [1], [2].

Aβ exists as soluble monomers and oligomers, and insoluble fibrils. Which of these forms of Aβ is the active species that are responsible for synaptic loss and neurodegeneration in AD is controversial [3], [4]. Neither monomeric nor fibrillar forms of Aβ appear to be responsible [4], [5]. Rather, a number of studies indicate that oligomeric Aβ is the most potent neurotoxic species in association with AD [6], [7], [8], [9], [10]. For example, oligomeric Aβ reduces neuronal viability approximately 10-fold more efficiently than fibrillar Aβ [11].

Aβ levels in healthy brain are delicately regulated by a dynamic equilibrium between production of Aβ from APP and clearance of Aβ via perivascular drainage or enzymatic degradation. The cytotoxic process of AD is closely linked to an imbalance between the production and clearance of Aβ [12]. Therefore, restoration of this balance by increasing the degradation of Aβ might be a valid therapeutic modality for the treatment of AD [13]. Several endogenous proteases can degrade Aβ. Among these, neprilysin (NEP) is considered to be the physiological regulator of the Aβ levels in the brain parenchyma [14], [15], [16]. Intracerebral injections of a recombinant lentivirus expressing human NEP reduce Aβ deposits and neurodegenerative alterations in a mouse model of amyloidosis [17]. Implantation of primary fibroblast cells that express a secreted form of human NEP also significantly reduces plaque burdens in the mouse brain [18]. Consistently, the genetic ablation of NEP in mice markedly increases Aβ levels in whole brain and plasma, increases plaque burdens in the hippocampus, and leads to the development of AD-like neuropathology [19]. Lentivirus-mediated long-term expression of NEP improves behavioral performances and ameliorates neurodegenerative pathology in APP mice [20]. However, the therapeutic potential of NEP is controversial as over-expression of NEP failed to reduce the toxic oligomeric Aβ levels nor improve cognitive deficits in AD mice although it did reduce plaque formation [21].

Nuclear inclusion a (NIa) of turnip mosaic virus is a cytosolic protease with a strict substrate specificity for the consensus sequence of Val-Xaa-His-Gln [22]. In an *in vitro* study, we demonstrated that NIa specifically cleaves Aβ, which contains the Val-His-His-Gln sequence near to its putative α-secretase cleavage site [23]. We further showed that lentivirus-mediated expression of NIa in the brain of AD mice significantly reduced Aβ pathology and improved behavioral deficits [23], [24].

Several lines of evidence have suggested that the progression of AD may be associated with mitochondrial dysfunction [25], [26]. Aβ inhibits import of nuclear-encoded mitochondrial proteins, and subsequently impairs mitochondrial functions and morphology [27]. In neurons, the overexpression of Aβ results in mitochondrial fragmentation and an abnormal subcellular distribution of mitochondria by evoking an imbalance between mitochondrial fusion and fission [28]. Furthermore, Aβ impairs oxidative phosphorylation and ATP production in transgenic AD mice [29].

Here, we compared the functions of NIa and NEP, and found that NIa, but not NEP, cleaved oligomeric Aβ and prevented Aβ-induced cytotoxicity and mitochondrial dysfunction in human neuroblastoma cells. By tracing exogenously added Aβ, we determined that NIa prevents localization of endocytosed Aβ to mitochondria. Our study suggests that disruption of Aβ trafficking to mitochondria via intracellular degradation of Aβ is a valuable approach for preventing Aβ-induced cytotoxicity.

## Results

### NIa, but not NEP, Cleaves Oligomeric Aβ *In vitro*

**Figure 1:**
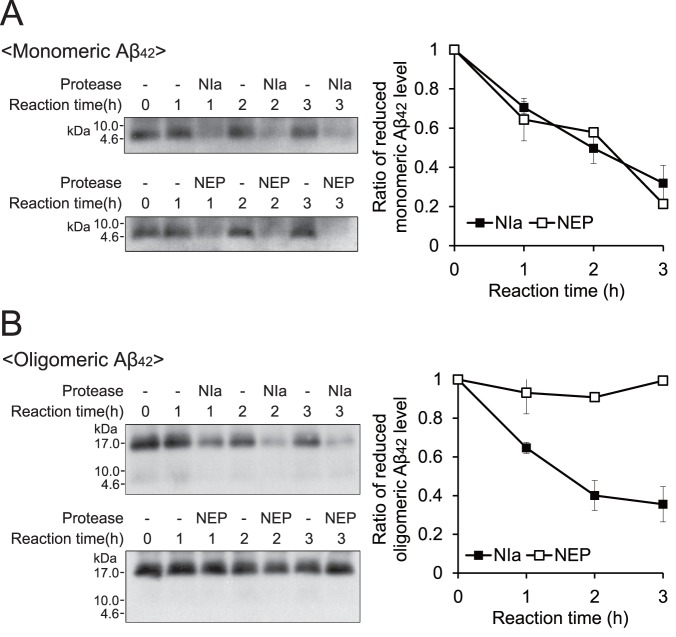
In vitro cleavage of Aβ by NIa and NEP. For the cleavage assay, 2.5 µM of monomeric (**A**) and oligomeric Aβ (**B**) were incubated with 0.5 µM of purified NIa or NEP for 1, 2, and 3 h. The reaction mixture was separated on a PeptiGel (Elpis Biotech), blotted, and probed with the anti-Aβ 6E10 antibody. The densities of the intact Aβ bands were quantified using NIH ImageJ software and plotted. Each data point and error bar represents the mean ± SD (n = 3).

We first performed an *in vitro* cleavage assay to compare the proteolytic activities of NIa and NEP for Aβ. Monomeric and oligomeric Aβ were incubated with the same amounts of purified NIa and NEP, and were then analyzed by Western blotting. Cleavage of Aβ was discerned by the disappearance of protein bands corresponding to intact monomeric and oligomeric Aβ. As expected, monomeric Aβ was efficiently cleaved by both NIa and NEP (Figure 1). However, oligomeric Aβ was only cleaved by NIa, not by NEP (Figure 1B). Notably, NIa cleaved both monomeric and oligomeric Aβ indistinguishably with a similar catalytic activity. To the best of our knowledge, NIa is the only cytosolic protease that can cleave both monomeric and oligomeric Aβ with a strict substrate specificity.

### NIa, but not NEP, Prevents Oligomeric Aβ-mediated Cytotoxicity

**Figure 2:**
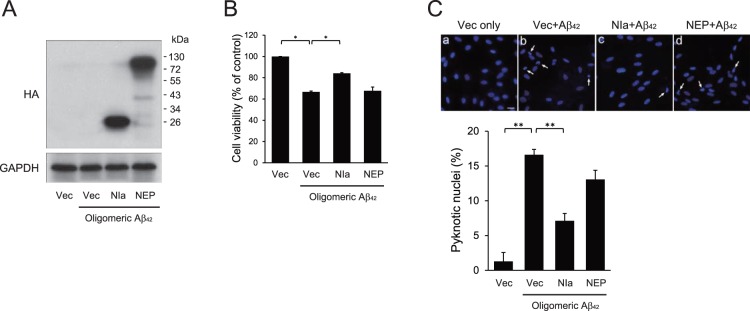
NIa, but not NEP, prevents Aβ-mediated cytotoxicity. Human neuroblastoma SH-SY5Y cells were transfected with pcDNA with no insert (Vec), or with pcDNA with cDNA encoding HA-NIa (NIa) or HA-NEP (NEP). After 24 h of incubation, cells were treated with 10 µM of oligomeric Aβ for an additional 48 h. (**A**) Western blotting with an anti-HA antibody showed that the expression levels of NIa and NEP were similar. GAPDH (detected with an anti-GAPDH antibody) was used as the loading control. (**B**) Cell viability was determined by using the MTT assay. Each bar and error bar represents the mean ± SD (n = 3); *p<0.05. (**C**) Cells were stained with Hoechst 33342 and viewed under a fluorescence microscope. Arrows indicate cells with pyknotic nuclei. The number of pyknotic nuclei was counted and plotted. Scale bar, 50 µm. Each bar and error bar represents the mean ± SD (n = 8); **p<0.01.

We next examined whether NIa or NEP can inhibit oligomeric Aβ-mediated cytotoxicity in human neuroblastoma SH-SY5Y cells. The cells were transformed with plasmids expressing HA-tagged NIa or NEP. Expression of NIa and NEP was assessed by Western blotting with an anti-HA antibody (Figure 2A). The amounts of plasmids used for cell transformations were adjusted so that the expression levels of NIa and NEP were almost equal in all the subsequent experiments.

Treatment of SH-SY5Y cells with oligomeric Aβ for 48 h reduced cell viability in a dose-dependent manner (Figure S1). The most prominent effects were seen with 10–20 µM Aβ. Thus, 10 µM oligomeric Aβ was used to observe the cytotoxic effects of Aβ in all the subsequent experiments. Under these conditions, Aβ reduced cell viability by ∼35% as assessed by 3-[4,5-dimethylthizaol-2-yl]-2,5-diphenyl tetrazolium bromide (MTT) assays (Figure 2B). This effect was significantly inhibited by NIa (∼16% reduction vs. control) but not by NEP (Figure 2B). Aβ-mediated cytotoxicity can also be monitored by nuclear fragmentation and condensation, a phenomenon known as pyknosis. Under control conditions, only 1–2% of cells underwent pyknosis as observed using a fluorescence microscope. In line with previous reports [30], [31], Aβ increased the percentage of pyknotic cells to ∼17%. This Aβ-mediated pyknosis was significantly reduced by NIa (∼7%), but not by NEP (Figure 2C). Collectively, these results indicate that NIa, but not NEP, prevents oligomeric Aβ-mediated cytotoxicity.

### NIa, but not NEP, Ameliorates Oligomeric Aβ-mediated Mitochondrial Dysfunction

**Figure 3:**
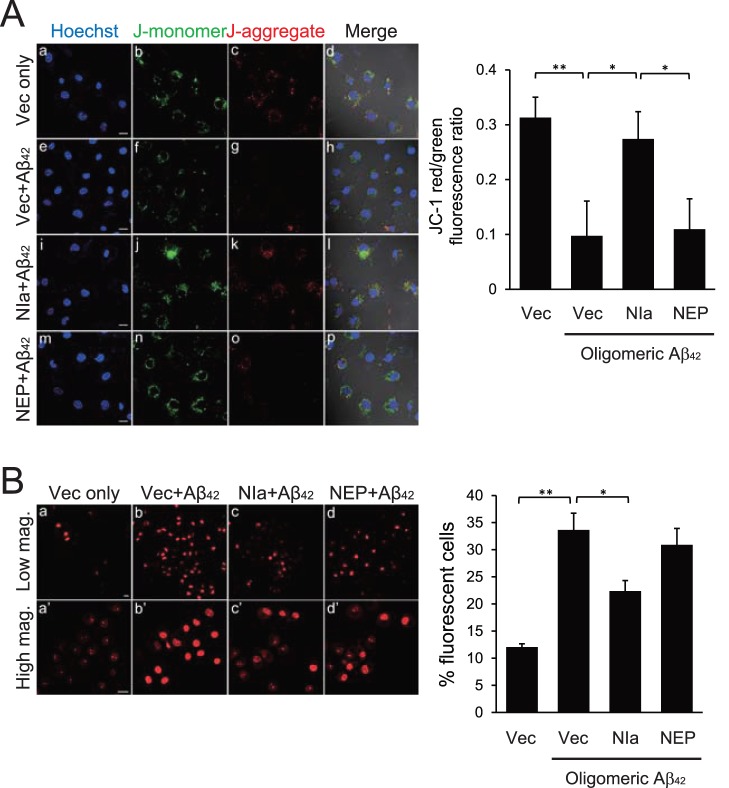
NIa, but not NEP, restores Aβ-mediated mitochondrial dysfunction. Human neuroblastoma SH-SY5Y cells were transfected with pcDNA with no insert (Vec), or with pcDNA with cDNA encoding HA-NIa (NIa) or HA-NEP (NEP). After 24 h of incubation, cells were treated with 10 µM of oligomeric Aβ for an additional 48 h. (**A**) Cells were treated with 2.5 µM of JC-1, an indicator of Ψm, for 15 min at 37°C and visualized by confocal microscopy. The intensities of red and green JC-1 fluorescence were quantitated using the MetaMorph imaging software and their ratios were plotted. Scale bar, 50 µm. Each bar and error bar represents the mean ± SD (n = 7); *p<0.05, **p<0.01. (**B**) Cells were incubated with 30 µM of DHE, an indicator of ROS for 30 min. Low (panels a–d) and high (panels a’–d’) magnification images of cells were obtained using a confocal microscope. Data represent the number of fluorescent cells as a percentage of the total number of cells in the observed field. Scale bar, 50 µm. Each bar and error bar represents the mean ± SD (n = 5); *p<0.05, **p<0.01.

Aβ reportedly is associated with mitochondrial dysfunction [27]. Thus, we examined whether NIa or NEP affect Aβ-induced mitochondrial dysfunction in SH-SY5Y cells. To monitor mitochondrial membrane potential (Ψm) using a confocal microscope, we utilized JC-1, which exists as a green-fluorescent J-monomer at depolarized membrane potentials and as a red-fluorescent J-aggregate at hyperpolarized membrane potentials. A decrease in the ratio of red fluorescence to green fluorescence indicates a decrease in Ψm. As expected, Aβ significantly reduced Ψm, which was reversed by NIa, but not by NEP (Figure 3A).

Next, we utilized the cell-permeable fluorescent dye dihydroethidium (DHE) to monitor the production of reactive oxygen species (ROS). When DHE is oxidized by superoxide anions to oxoethidium, it intercalates into DNA and generates red fluorescence [32]. In line with previous reports [33], Aβ increased the percentage of cells with red fluorescence (∼33% vs. ∼12% in control), which was significantly attenuated by NIa (∼21%), but not by NEP (Figure 3B). Taken together, these data indicate that NIa, but not NEP, ameliorates Aβ-mediated mitochondrial dysfunction.

### NIa, but not NEP, Prevents Accumulation of Aβ in Mitochondria

**Figure 4:**
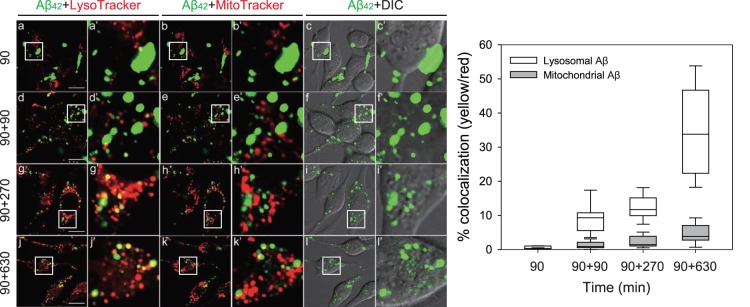
Endocytosed oligomeric Aβ accumulates in lysosomes and mitochondria. Human neuroblastoma SH-SY5Y cells were treated with 2.5 µM of Alexa Fluor-labeled Aβ oligomers for 90 min (pulse) and were further incubated in fresh media for 90, 270, or 630 min (chase). Cells were co-stained with LysoTracker and MitoTracker, and observed under a confocal microscope. The images indicated by the open boxes are shown in a higher magnification in the adjacent columns. The yellow color in the merged image indicates co-localization of green (Alexa Fluor 488-labeled Aβ) and red (LysoTracker, Red/MitoTracker, Deep Red) fluorescence. The percentages of lysosomes (white bars) or mitochondria (gray bars) that co-localized with Aβ were plotted. Scale bar, 50 µm. Each bar and error bar represents the mean ± SD (n = 10). DIC, differential interference contrast.

We next sought to elucidate how NIa prevents Aβ-mediated mitochondrial dysfunction. To this end, we traced the intracellular trafficking of exogenously added Aβ in SH-SY5Y cells. Oligomeric Aβ was labeled with Alexa Fluor 488 and was then added to the culture media. After 90 min of incubation (pulse), the culture media was replaced with fresh media not containing Aβ and was then further incubated for 90, 270, and 630 min (chase). The cells were then fixed and co-stained with LysoTracker and MitoTracker. After 90 min of chase, ∼10% of LysoTracker, but none of the MitoTracker, co-localized with the Alexa Flour. This suggested that the majority of the exogenously added Aβ was present in endosomes, some was present in lysosomes, and none was present in mitochondria at this stage. However, the percentage of LysoTracker or MitoTracker that co-localized with Alexa fluorescence gradually increased as the duration of the chase increased. After 630 min of chase, ∼35% of LysoTracker and ∼5% of MitoTracker co-localized with the Alexa Flour (Figure 4). These results are consistent with earlier observations that exogenously added Aβ reaches lysosomes via endocytosis, where a portion of the peptide enters mitochondria; however, the mechanism is unknown.

**Figure 5:**
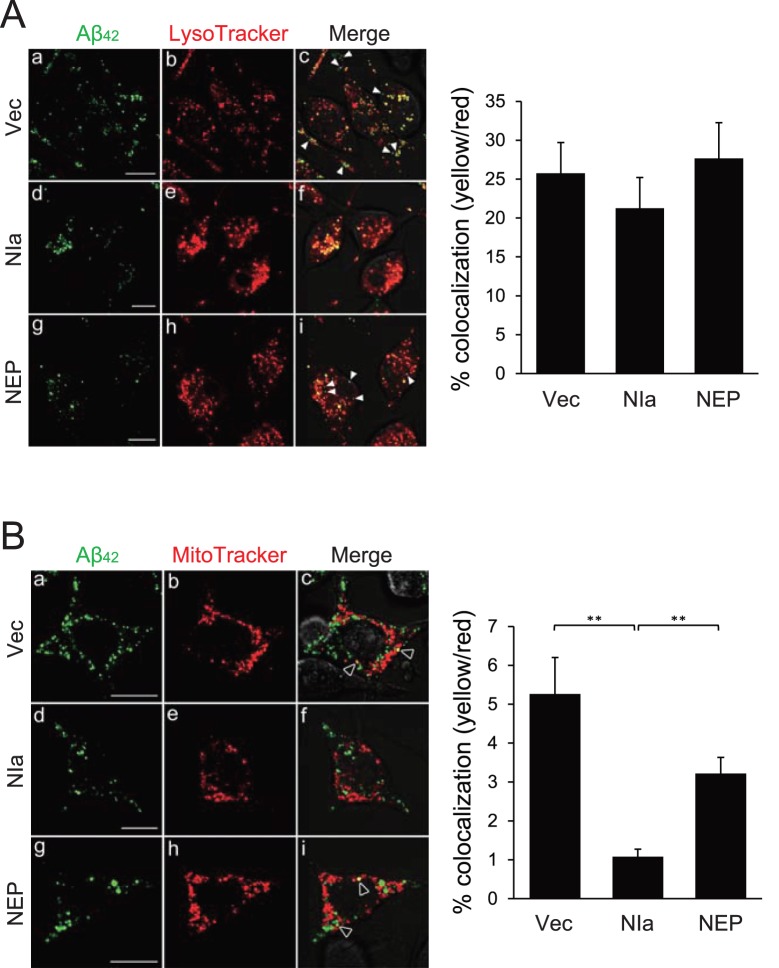
NIa, but not NEP, prevents accumulation of Aβ in mitochondria. Human neuroblastoma SH-SY5Y cells were transfected with pcDNA with no insert (Vec), or with pcDNA with cDNA encoding HA-NIa (NIa) or HA-NEP (NEP). After 24 h of incubation, cells were treated with 2.5 µM of Alexa Fluor 488-labeled Aβ oligomers for an additional 18 h. Cells were stained with LysoTracker (**A**) or MitoTracker (**B**), and observed by confocal microscopy. White arrowheads in panel A indicate Aβ that co-localized with lysosomes and open arrowheads in panel B indicate Aβ that co-localized with mitochondria. The percentages of lysosomes or mitochondria that co-localized with Aβ were plotted. Scale bar, 50 µm. Each bar and error bar represents the mean ± SD (n = 10); **p<0.01.

We next examined whether NIa or NEP affect trafficking of oligomeric Aβ to mitochondria. SH-SY5Y cells transformed with plasmids expressing NIa or NEP were treated with Alexa Flour 488-labeled oligomeric Aβ for 18 h. At this time point, most of the Alexa Flour co-localized with either LysoTracker or MitoTracker. Under normal conditions, ∼25% and ∼5% of LysoTracker and MitoTracker, respectively, co-localized with the Alexa Flour. Neither NIa nor NEP significantly affected the co-localization of LysoTracker with the Alexa Flour (Figure 5A). However, NIa, but not NEP, significantly reduced the percentage of MitoTracker that co-localized with the Alexa Flour (∼1%) (Figure 5B). Co-localization of MitoTracker and the Alexa Flour was further confirmed by 3-dimensional reconstruction of the confocal images (Figure S2, Video S1–4). The Alexa Flour represents intact Aβ after incubation for 18 h, supporting the validity of our experimental approach (Figure S3). Collectively, these data suggest that NIa prevents the accumulation of oligomeric Aβ in mitochondria by proteolytically degrading the peptide in the cytosol.

## Discussion

The NIa protease of turnip mosaic virus has a strict substrate specificity for the consensus sequence of Val-Xaa-His-Gln [22]. This protease is involved in the cleavage of viral polyproteins to generate mature viral proteins. We noticed the same Val-His-His-Gln consensus sequence in Aβ near to its putative α-secretase cleavage site and surmised that this sequence could be cleaved by NIa. Indeed, NIa specifically cleaves Aβ *in vitro* and significantly reduces Aβ-induced cell death in rat neuroblastoma cells [23]. Furthermore, lentivirus-mediated expression of NIa in the brain of AD mice significantly reduces cerebral Aβ levels and plaque depositions, and recovers behavioral deficits [24]. These results raised the possibility that NIa can be used as a therapeutic modality for the treatment of AD.

Currently, more than 20 endogenous Aβ-cleaving enzymes have been identified [34]. Among them, NEP is considered to have a major role in the metabolism of Aβ in the brain. The possible therapeutic use of NEP for AD was proposed because NEP ameliorates neurodegenerative pathology and also improves behavioral performances in APP mice [20]. However, this earlier enthusiasm has been challenged. For examples, over-expression of NEP does not improve cognitive deficits in AD mice [21]. This might be explained, at least partially, because NEP cannot cleave the more toxic oligomeric Aβ, as shown here. In addition, it should be noted that NEP has diverse physiological roles in the brain. For example, overexpression of NEP causes a reduction in cAMP-responsive element-binding protein-mediated transcription, age-dependent axon degeneration, and premature death in flies [35]. Sustained NEP activation may also be detrimental in mammals because NEP can degrade a wide range of circulating peptides, including enkephalin, atrial natriuretic peptide, endothelin, and substance P [36]. Therefore, NIa has certain advantages over NEP as a therapeutic modality for AD with its unique capability of cleaving the more toxic oligomeric Aβ and its relatively high substrate specificity.

The controversy surrounding the molecular mechanism underlying the cytotoxicity of Aβ in brains has yet to be settled. Among the several hypotheses, one suggests that Aβ exerts its detrimental effects partly by interfering with mitochondrial functions. Aβ is internalized via raft-mediated endocytosis [37]. The internalized Aβ reaches the mitochondria, where it binds to a mitochondrial enzyme called Aβ-binding alcohol dehydrogenase (ABAD). It remains to be seen how the endocytosed, thus intraluminal, Aβ reaches the mitochondria. The interaction between Aβ and ABAD promotes leakage of ROS, mitochondrial dysfunction, and cell death [38]. Furthermore, inhibition of the Aβ-ABAD interaction using a decoy peptide improves mitochondrial function in AD mice [39]. NIa did not interfere with the internalization of Aβ or with the transport of Aβ to lysosomes, but reduced the amounts of Aβ localized in mitochondria (Figure 5). Therefore, it appears that NIa cleaves Aβ that was in transit from lysosomes to mitochondria. Our chase experiments suggest that Aβ travels through endosomes and lysosomes and that a portion of Aβ further travels to mitochondria (Figure 4). Considering that NIa functions primarily in the cytosol, it is possible that Aβ transiently passes the cytosol during transit from the lysosomes to mitochondria. However, we could not definitively test this hypothesis due to limitations in current imaging techniques. Interpretation of our data is also partially hampered by the fact that the traffic routes allowing the localization of Aβ in mitochondria are largely unknown.

Collectively, we demonstrated that NIa prevents Aβ-mediated cytotoxicity and associated mitochondrial dysfunction by reducing the amounts of Aβ localized in the mitochondria. During the pathogenesis of AD, the route linking lysosomes to mitochondria can be viewed as a “Thermopylae pass”. Annihilation of the invading Aβ at this “pass” can be a winning strategy in the battle against AD.

## Materials and Methods

### Reagents and Materials

Synthetic Aβ_42_ peptide was purchased from Anygen (Gwangju, Korea). Active recombinant human NEP was from Enzo Life Science International (Farmingdale, NY, USA). Mouse monoclonal anti-Aβ antibody against residues 1–16 (6E10) was purchased from Covance (Princeton, NJ, USA). DHE was from Sigma-Aldrich (St Louis, MO, USA). JC-1, LysoTracker Red DND-99, and MitoTracker Deep Red FM were from Molecular Probes (Eugene, OR, USA). Unless otherwise noted, all other chemicals and reagents were purchased from Sigma.

### Preparation of Aβ_42_ Oligomer

Aβ_42_ oligomers were prepared according to the method described by Stine *et al.* [40]. Synthetic Aβ_42_ peptides were initially solubilized in 1,1,1,3,3,3-hexafluoroisopropanol (Fluka) to a concentration of 1 mM, to monomerize pre-existing aggregates. Following evaporation of the 1,1,1,3,3,3-hexafluoroisopropanol in a fume hood overnight, the resulting peptide film was stored desiccated at −20°C. Subsequently, the peptide was resuspended in anhydrous dimethyl sulfoxide to a concentration of 2.5 mM and bath sonicated for 10 min. To enrich oligomers, phenol-red free Dulbecco’s modified Eagle’s medium (DMEM; Gibco) was added under continuous vortexing to bring the peptide to a final concentration of 100 µM and incubated at 4°C for 24 h.

### Purification of the NIa Protease and *In vitro* Cleavage Assay

Purification of the NIa protease was performed as described by Han *et al.* [23]. For the *in vitro* cleavage assay, 0.5 µM of purified NIa or recombinant NEP was incubated with 2.5 µM of monomeric or oligomeric Aβ in a time-dependent manner. The buffers used in this reaction were as follows: NIa (20 mM HEPES [pH 7.4], 10 mM KCl, 10 mM MgCl_2_) and NEP (50 mM Tris-HCl [pH 9.0], 0.05% Brij35). After incubation, the reaction mixture was separated on a PeptiGel (Elpis Biotech), blotted, and probed with the anti-Aβ 6E10 antibody [41].

### Cell Culture and DNA Transfection

Human neuroblastoma SH-SY5Y cells were grown in DMEM (Hyclone) supplemented with 10% fetal bovine serum (Hyclone), 100 U/ml penicillin, and 100 µg/ml streptomycin (Invitrogen). Cells were transiently transfected with plasmid DNA using Lipofectamine LTX (Invitrogen) according to the manufacturer’s instructions. To express the turnip mosaic virus NIa and the NEP protease in mammalian cells, codon-optimized NIa and NEP genes were subcloned into the pcDNA3 vector (Invitrogen) containing an N-terminal HA tag. A matching vector without an insert was used as a control.

### Quantification of Cell Death/Survival

MTT (Sigma) was dissolved in phosphate buffered saline at a concentration of 2.5 mg/ml. A volume of MTT solution equivalent to 20% of the culture media volume was added to the cell culture at 37°C for 2 h. A volume of dimethyl sulfoxide (solubilization solution) equivalent to the culture media volume was added, and cells were placed on a shaker until the resulting formazan crystals were completely dissolved. The absorbance of the samples was measured at 570 nm, and the background absorbance of each well was measured at 690 nm. SH-SY5Y cells were examined for pyknotic nuclei by Hoechst 33342 staining following the methods described by Wyttenbach *et al.* [42] and Sellamuthu *et al.* [43].

### Measurement of Ψm

Ψm was determined by staining SH-SY5Y cells with JC-1 and was measured by confocal microscopy. SH-SY5Y cells were cultured on poly-L-lysine-coated coverslips. After exposure to Aβ, cells were incubated in DMEM containing 2.5 µM JC-1 for 15 min at 37°C. The cells were washed and fluorescent images were then obtained immediately using a Fluoview FV 1000 confocal laser scanning microscope. Data were analyzed with MetaMorph imaging software to quantify the intensities of red and green fluorescence. The results were expressed as the ratio of red fluorescence to green fluorescence.

### Measurement of ROS Production

ROS production in SH-SY5Y cells was assayed using the oxidative fluorescent dye DHE. SH-SY5Y cells were cultured on poly-L-lysine-coated coverslips. After exposure to Aβ, cells were loaded with 30 µM of DHE for 30 min at 37°C. The cells were washed to remove excess DHE and fluorescent images were captured immediately using a Fluoview FV 1000 confocal laser scanning microscope. The excitation and emission wavelengths were 510 nm and 590 nm, respectively. Images were analyzed using MetaMorph imaging software. The number of fluorescent cells were counted and represented as a percentage of the total number of cells in each image field.

### Labeling of Aβ_42_ Oligomers

The labeling reaction was performed using the Alexa Fluor 488 Microscale Protein Labeling Kit (Invitrogen). The procedure was described by Jungbauer *et al.* [44] in detail.

### Cellular Uptake of Alexa Fluor 488-labeled Aβ_42_ Oligomers

SH-SY5Y cells were cultured on poly-L-lysine-coated coverslips for 24 h. The cells were treated with 2.5 µM of Alexa Fluor 488-labeled Aβ_42_ oligomers in phenol-red-free DMEM (Gibco) supplemented with 1% N2 supplement (Invitrogen), 100 U/ml penicillin, and 100 µg/ml streptomycin (Invitrogen) for a further 90 min. At the end of the treatment, cells were further incubated for various lengths of time. Cells grown in the presence of Alexa Fluor 488-labeled Aβ_42_ oligomers were stained with 200 nM of LysoTracker Red and 100 nM of MitoTracker Deep Red in phenol-red-free DMEM for 30 min at 37°C. The cells were washed extensively and were then visualized immediately using a Fluoview FV 1000 confocal laser scanning microscope equipped with 100× and 60× oil-immersion objectives and capable of additional 3–4× zoom.

### Statistical Analysis

Results are expressed as the means ± standard deviation (SD). Comparisons between two groups were performed using the Student’s *t*-test. Comparisons between multiple groups were performed by one-way ANOVA with the Bonferroni correction. Statistical analyses were conducted with StatView software version 5.0 (SAS Institute Inc.). A *p*-value of less than 0.05 was considered statistically significant.

## Supporting Information

Figure S1 **Dose-dependent effects of oligomeric Aβ on cell viability.** SH-SY5Y cells were incubated with various concentrations of oligomeric Aβ for 48 h. Cell viability was determined by using the MTT assay. Each bar and error bar represents the mean ± SD (n = 4); **p<0.01.
(EPS)

Figure S2 **Assessment of mitochondrial accumulation of Aβ by confocal microscopy.** SH-SY5Y cells were treated with 2.5 µM of Alexa Fluor-labeled Aβ oligomers for 90 min and were further incubated in fresh media for 630 min. Cells were stained with MitoTracker and observed under a laser scanning confocal microscope. (**A**) Reconstruction of 3-D images was performed with 50–60 Z-directional slices (0.1 µm thick) of the confocal images. The 3-D images were then virtually re-sliced in YZ axis (marked by white broken lines) to obtain transversal images (**a_1_, a_2_**). Open arrowheads indicate Aβ that co-localized with mitochondria. Note that all the 2 yellow dots seen in XY planes (**a**) are also yellow when observed in YZ planes (**a_1_, a_2_**). Scale bar, 20 µm. (**B**) The images of the individual Z slices were arranged by their positions along the Z-axis from top to bottom. Arrows in images #6–11 indicate the Aβ fluorescence shown in panel a_1_, and arrows in images #19–23 indicate the Aβ fluorescence shown in panel a_2_.
(TIF)

Figure S3 **Fluorescence of Alexa Fluor-labeled Aβ represents intact Aβ in SH-SY5Y cells.** SH-SY5Y cells were treated with 2.5 µM of Alexa Fluor-labeled Aβ (green) for 90 min and were further incubated in fresh media for 18 h. Cells were stained with MitoTracker Red CMXRos (red) and fixed with methanol for 4 min. Aβ was detected either by fluorescence of Alexa Fluor (green) or by immunostaining with the 6E10 antibody (blue). (**A**) Images with low (panels a–e) and high (panels a’–e’) magnifications were obtained using a confocal microscope. A filled arrow in merged images indicates Aβ colocalized with MitoTracker, whereas open arrows indicate Aβ not colocalized with MitoTracker. Scale bars, 10 µm. (**B**) Pair-wise merged images were created using the images shown in panel A. Merge 1 shows that all Alexa signals are overlapped with 6E10 signals. Merge 2 and 3 show that one of the Aβ signal is colocalized with MitoTracker. Scale bars, 10 µm.
(PDF)

Video S1 **360-degree view of the reconstituted 3-D confocal images.** Video was created for the positive dot that was shown in Figure S2 (panel a_1_).
(AVI)

Video S2 **360-degree view of the reconstituted 3-D confocal images.** Video was created for the positive dot that was shown in Figure S2 (panel a_2_).
(AVI)

Video S3 **360-degree view of the reconstituted 3-D confocal images.** Video was created for one of the negative dots that were shown in Figure S2 (panel a).
(AVI)

Video S4 **360-degree view of the reconstituted 3-D confocal images.** Video was created for one of the negative dots that was shown in Figure S2 (panel a).
(AVI)
