# Supplementary figures and images for: Intracellular Cleavage of Amyloid β by a Viral Protease NIa Prevents Amyloid β-Mediated Cytotoxicity
Source: PLoS One. 2014 Jun 10;9(6):e98650. doi: 10.1371/journal.pone.0098650 (PMC4051590; doi:10.1371/journal.pone.0098650)

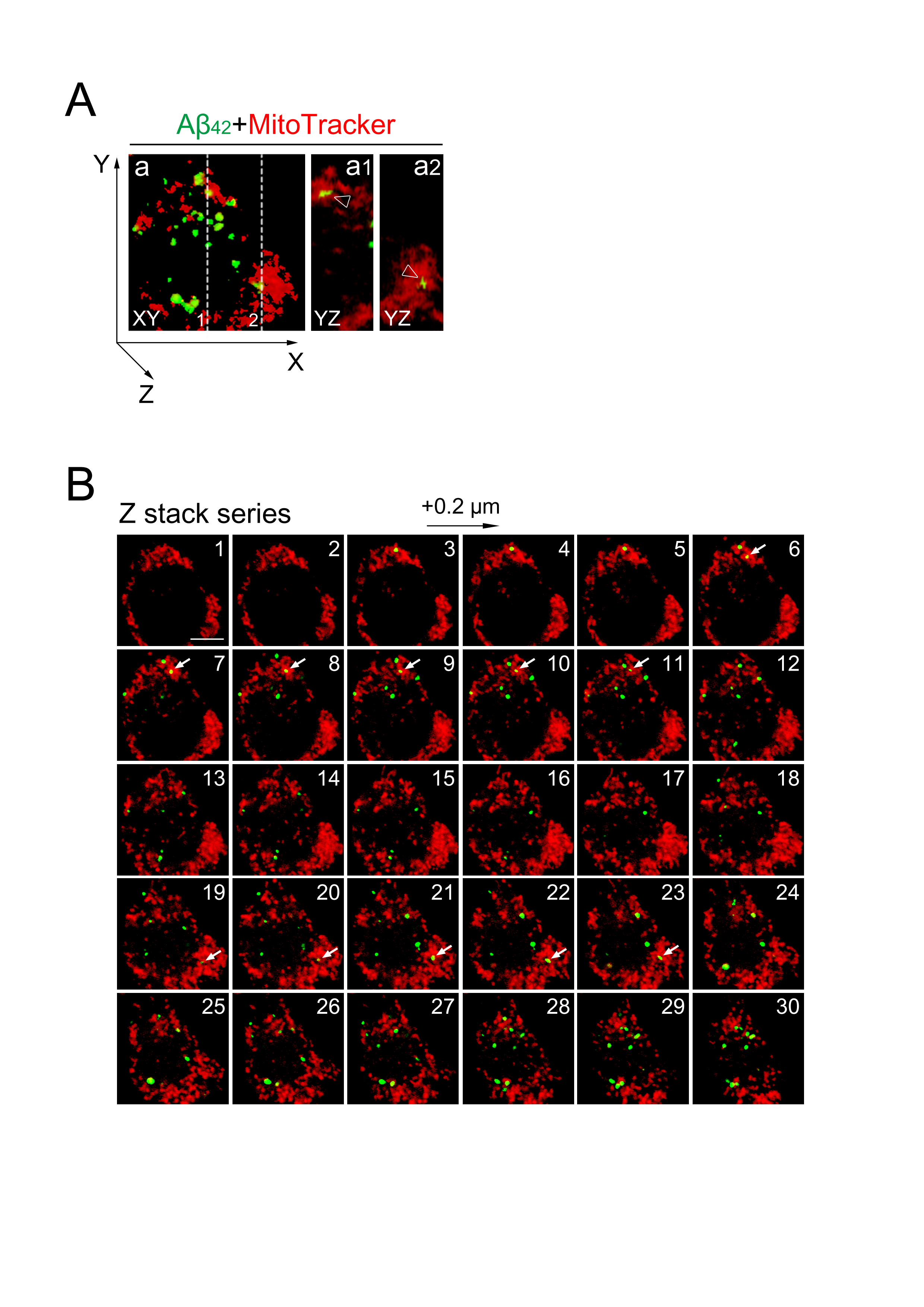

Supplement: Figure S2 — Assessment of mitochondrial accumulation of Aβ by confocal microscopy. SH-SY5Y cells were treated with 2.5 µM of Alexa Fluor-labeled Aβ oligomers for 90 min and were further incubated in fresh media for 630 min. Cells were stained with MitoTracker and observed under a laser scanning confocal microscope. (A) Reconstruction of 3-D images was performed with 50–60 Z-directional slices (0.1 µm thick) of the confocal images. The 3-D images were then virtually re-sliced in YZ axis (marked by white broken lines) to obtain transversal images (a1, a2). Open arrowheads indicate Aβ that co-localized with mitochondria. Note that all the 2 yellow dots seen in XY planes (a) are also yellow when observed in YZ planes (a1, a2). Scale bar, 20 µm. (B) The images of the individual Z slices were arranged by their positions along the Z-axis from top to bottom. Arrows in images #6–11 indicate the Aβ fluorescence shown in panel a1, and arrows in images #19–23 indicate the Aβ fluorescence shown in panel a2. (TIF) [file pone.0098650.s002.tif]

**A**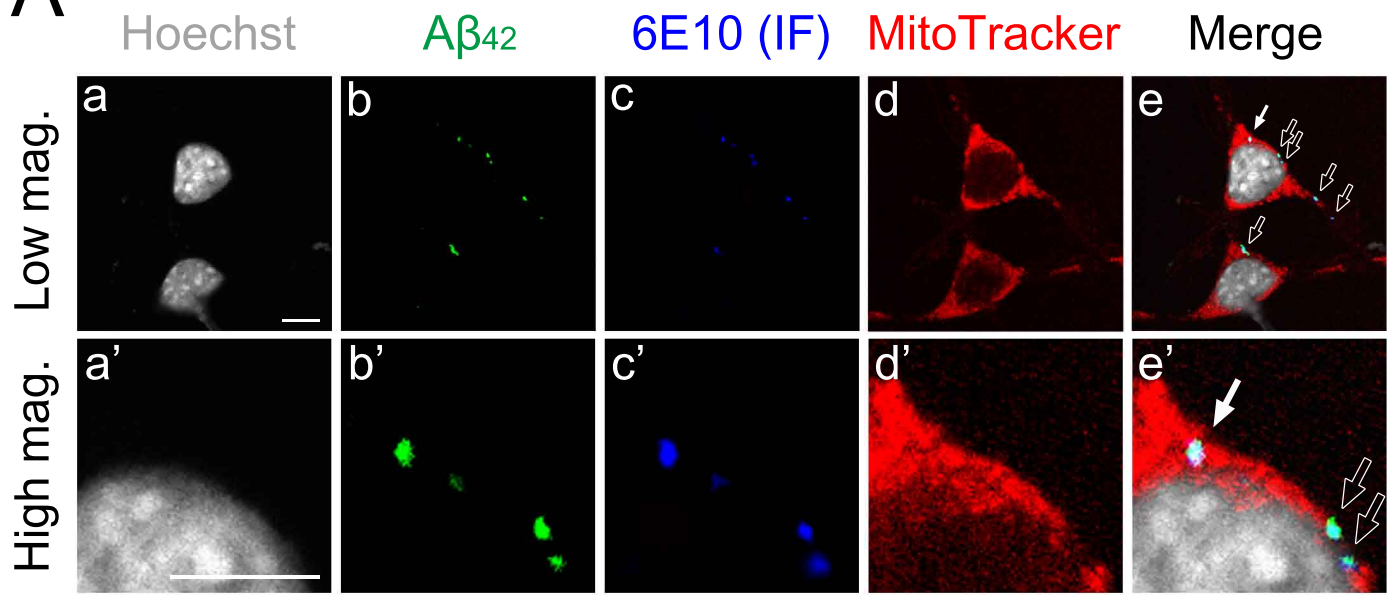**B**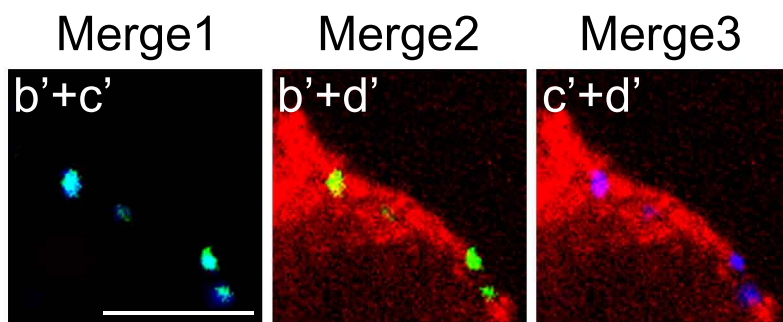

Supplement: Figure S3 — Fluorescence of Alexa Fluor-labeled Aβ represents intact Aβ in SH-SY5Y cells. SH-SY5Y cells were treated with 2.5 µM of Alexa Fluor-labeled Aβ (green) for 90 min and were further incubated in fresh media for 18 h. Cells were stained with MitoTracker Red CMXRos (red) and fixed with methanol for 4 min. Aβ was detected either by fluorescence of Alexa Fluor (green) or by immunostaining with the 6E10 antibody (blue). (A) Images with low (panels a–e) and high (panels a’–e’) magnifications were obtained using a confocal microscope. A filled arrow in merged images indicates Aβ colocalized with MitoTracker, whereas open arrows indicate Aβ not colocalized with MitoTracker. Scale bars, 10 µm. (B) Pair-wise merged images were created using the images shown in panel A. Merge 1 shows that all Alexa signals are overlapped with 6E10 signals. Merge 2 and 3 show that one of the Aβ signal is colocalized with MitoTracker. Scale bars, 10 µm. (PDF) [file pone.0098650.s003.pdf]
